# The impact of weight loss interventions on thyroid function: a systematic review and meta-analysis

**DOI:** 10.1097/MS9.0000000000003428

**Published:** 2025-05-26

**Authors:** Sandeep Samethadka Nayak, Seyyed Mohammad Hashemi, Masum Patel, Nimra Shafi, Pubali Biswas, Sepide Javankiani, Padmavathi Jaladi, Sanjana B. Patil, Rahiq Rashid, Ehsan Amini-Salehi, Daniyal Ameen, Khushbu Viresh Janani, Anil Kumar Jonnalagadda, Kwame Boateng Agyeman

**Affiliations:** a Division of Hospital Medicine, Department of Internal Medicine, Bridgeport Hospital, Yale New Heaven, Bridgeport, CT, USA; b Cardiovascular Research Center, Hormozgan University of Medical Sciences, Bandar Abbas, Iran; c Byramjee Jeejeebhoy Medical College 34, Jalaramnagar Society, Mehsana, Gujarat, India; d Department of Medicine, Arnot Ogden Medical Center, Elmira, NY, USA; e Vydehi Institute of Medical Sciences and Research Centre, Nallurahalli Main Road, Whitefield, Bengaluru, Karnataka, India; f General Surgery Department, Tehran University of Medical Sciences, Tehran, Iran; g Molecular and Cellular Biology, University of Connecticut Honors College, Mansfield Rd, USA; h Vydehi Institute of Medical Sciences and Research Centre, Bengaluru, Karnataka, India; i Gastrointestinal and Liver Diseases Research Center, Guilan University of Medical Sciences, Rasht, Iran; j Department of Internal Medicine, Yale New Haven Health Bridgeport Hospital, USA; k Soundview Medical Associates, Department of Internal Medicine, Hartford Healthcare, Wilton, CT, USA; l Department of Cardiology, John Peter Smith Hospital, Texas, USA; m Division of General Medicine, Department of Medicine, Loma Linda University Health, California, USA

**Keywords:** bariatric surgery, BMI reduction, FT3 levels, FT4 levels, obesity, thyroid hormones, weight loss

## Abstract

**Background:**

This study explores how weight loss impacts thyroid hormone levels, particularly free T3 (FT3) and FT4, in obese individuals, given the key role these hormones play in regulating metabolism and gene expression.

**Methods:**

A comprehensive literature search in PubMed, ISI/Web of Science, and Scopus databases identified studies examining the correlation between weight, body mass index (BMI), and thyroid hormone levels.

**Results:**

The analysis revealed that BMI reduction significantly decreases FT3 levels [odds ratio (OR) = 2.31, 95% confidence interval (CI): 1.73–3.10, *P* < 0.01] and increases FT4 levels (OR = 0.68, 95% CI: 0.47–0.98, *P* = 0.04). Weight loss results in a significant reduction in FT3 (OR = 2.47, 95% CI: 1.68–3.64, *P* < 0.01) and thyroid-stimulating hormone (TSH) (OR = 1.76, 95% CI: 1.15–2.69, *P* < 0.01), as well as a significant increase in FT4 (OR = 0.69, 95% CI: 0.55–0.88, *P* < 0.01). Subgroup analysis indicated that changes in thyroid hormone levels were more pronounced in obese individuals who underwent diet calorie restriction compared to bariatric surgery.

**Conclusion:**

The study results indicate that weight reduction leads to a decrease in TSH and FT3 levels and an increase in FT4 levels. Calorie restriction through diet has a more pronounced effect on thyroid function compared to bariatric surgery. Clinicians should approach elevated TSH levels in obese patients with caution, emphasizing weight management strategies as the primary therapeutic intervention before resorting to pharmacological treatments.

## Graphical abstract

**Figure:**
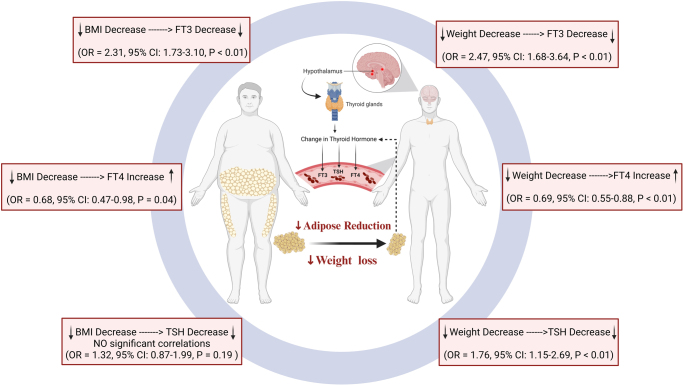

## Introduction

Obesity has emerged as a significant global public health issue^[1–4]^. Research has demonstrated a correlation between body weight and thyroid function, indicating that obesity can increase the risk of developing overt or subclinical hypothyroidism (SCH)^[5]^. The initial treatment approach for obesity is behavior modification, which involves limiting dietary intake^[6]^. Another alternative is bariatric surgery^[7]^. Losing weight can improve irregularities in blood glucose and lipids, reduce the body’s inflammatory state, and safeguard thyroid function^[8,9]^.

**HIGHLIGHTS**

- Obesity significantly impacts thyroid function, influencing thyroid hormone levels, yet the relationship between weight loss and thyroid function is not well understood.
- The meta-analysis revealed that weight loss significantly decreases free T3 (FT3) and thyroid-stimulating hormone levels while increasing FT4 levels.
- Weight management should be integrated into treatment plans for hypothyroid patients, particularly those undergoing bariatric surgery, with careful monitoring and adjustment of thyroid hormone therapies post-weight loss.

Thyroid hormone (TH) regulates metabolic pathways crucial for proper growth and development^[10]^. TH, released as thyroxine (T4) and triiodothyronine (T3), circulates in the bloodstream for a few hours to days before being metabolized by deiodinase enzymes or redistributed. Thyroid-stimulating hormone (TSH) is vital in assessing thyroid function due to its negative feedback mechanism via the hypothalamic–pituitary axis. In the blood, T3 and T4 are primarily bound to proteins such as thyroxine-binding globulin (TBG), transthyretin, and albumin. Only a small percentage of these hormones are unbound and physiologically active, known as free T3 (FT3) and FT4^[11,12]^.

These free hormones easily enter cells and bind to nuclear thyroid receptors (TRs), regulating gene transcription involved in metabolism, cellular differentiation, and growth. This process affects metabolic rate, protein synthesis, and catecholamine sensitivity^[13]^. TH plays a central role in energy expenditure and body weight balance^[10]^. The influence of thyroxine levels on weight has been extensively studied, reinforcing our understanding of the effects of FT3 and FT4 at both cellular and physiological levels^[14]^. While it is well established that hyperthyroidism or hypothyroidism affects weight, the inverse relationship – how weight alterations affect TH levels – is less clear and requires further exploration. A decrease in weight can impact the therapeutic levels of thyroid medications. For instance, weight gain might necessitate an increase in medication dosage to maintain appropriate levels of T3 and T4. Conversely, weight loss might require a reduction in dosage to avoid symptoms of hyperthyroidism^[15]^.

Several possible mechanisms have been proposed regarding the effect of obesity on thyroid function^[16,17]^. Recent studies have shown conflicting or insignificant results regarding the effect of weight loss on TH levels^[18,19]^. This is pertinent because obesity and its therapeutic options have become increasingly common and their impact on thyroid function needs to be clarified further. This study aims to determine how weight loss affects TH levels in order to help clinicians understand thyroid function in various interventions, such as lifestyle changes or surgical approaches.

## Method

### Setting

This systematic review and meta-analysis was conducted to examine how weight reduction influences thyroid function. The study was reported adhering to the guidelines of the Preferred Reporting Items for Systematic Reviews and Meta-Analyses (PRISMA) and AMSTAR (Assessing the Methodological Quality of Systematic Reviews) guidelines^[20,21]^.

### Study selection

On 20 April 2024, comprehensive searches of three major databases (PubMed, ISI/WoS, and Scopus) were conducted by two independent researchers using keywords such as “Thyroid Diseases,” “Thyroid Hormones,” “Thyroid Stimulating Hormone,” “Thyrotropin,” “Triiodothyronine,” “Body Weight Change,” and “Weight Loss.” Additionally, the references of the included studies were scrutinized to identify any potentially overlooked research. The detailed search strategies and specific keywords employed for each database are documented in Table S1, http://links.lww.com/MS9/A837S.

### Inclusion and exclusion criteria

Because the aim of the study was to assess how weight loss can impact TH levels, only longitudinal studies that followed patients over time were included. Observational studies (e.g., cross-sectional and case-control) were excluded. Additionally, exclusion criteria encompassed studies that assessed the impact of thyroid function on anthropometric indices, as well as systematic reviews, meta-analyses, narrative reviews, randomized clinical trials, editorials, and commentaries. Studies involving pregnant populations were also omitted.

### Quality assessment

Using the Joanna Briggs Institute Critical Appraisal Checklist^[22]^, two researchers evaluated the included studies’ quality independently. There are several questions on this checklist, and the answers can be classified as yes, no, uncertain, or not relevant. Table 1 summarizes the overall quality scores; Table S2, http://links.lww.com/MS9/A837S, provides more specific details.

**Table 1:** Characteristics of the included studies

Name of first author	Year of publication	Population	Intervention	Duration of follow-up	centerTotal sample size (male/female)	Age of population (Mean ± SD)	Country	Reported correlation between anthropometric indices/thyroid hormone	Quality of the study based on the JBI checklist
Abdelbaki^[23]^	2019	Adult obese	Surgery	12 months	482 (NA)	NA	Egypt	BMI and TSH	10/11
								BMI and FT4	
								BMI and FT3	
Aeberli^[24]^	2010	Children obese	Dietary modification	2 months	206 (119/87)	14.1 ± 1.9	Switzerland	BMI and TSH	10/11
								BMI and FT4	
Calapkorur^[25]^	2020	Adult obese	Surgery	3 months	20 (0/20)	57 ± 40.15	Turkey	BMI and TSH	10/11
								Weight and TSH	
Chen^[26]^	2022	Adult obese	Surgery	12 months	85 (33/52)	27.8 ± 8.0	China	BMI and TSH	10/11
								BMI and FT4	
								BMI and FT3	
Dall’Asta^[27]^	2010	Adult obese	Surgery	6 months	258 (42/216)	41.1 ± 0.04	USA	BMI and FT4	11/11
								BMI and FT3	
Ekinci ^[28]^	2015	Children obese	Dietary modification	12 months	126 (66/60)	8.5 ± 2.1	Turkey	BMI and TSH	11/11
Janssen^[29]^	2014	Adult subclinical hypothyroidism	Surgery	12 months	61 (NA)	42 ± 13	Netherlands	BMI and TSH	10/11
Kamal^[30]^	2023	Adults	Surgery	12 months	106 (12/94)	42 ± 6.1	Egypt	BMI and TSH	10/11
Karaman^[31]^	2020	Adults	Surgery	12 months	106 (20/86)	36.4 ± 11.3	Turkey	BMI and TSH	11/11
								BMI and FT4	
								BMI and FT3	
								Weight and TSH	
								Weight and FT4	
								Weight and FT3	
Kim^[32]^	2023	Adults	Dietary modification	3 months	49 (38/11)	43.9 ± 6.2	South Korea	BMI and TSH	11/11
								Weight and TSH	
Licenziati^[33]^	2019	Children/adolescents obese	Dietary modification	12 months	116 (57/59)	11.1 ± 2.4	Italy	BMI and TSH	11/11
Liu^[34]^	2017	Adults obese	Dietary modification	6 − 24 months	484 (NA)	51.6 ± 9.0	USA	Weight and TSH	11/11
								Weight and FT4	
								Weight and FT3	
Moraes^[35]^	2005	Adults obese	Surgery	12 months	72 (10/62)	39.6 ± 9.8	Brazil	BMI and TSH	10/11
Mwafya^[36]^	2017	Adults obese	Dietary modification	6 months	94 (0/94)	30.12 ± 9.3	Palestinian	BMI and TSH	9/11
								BMI and FT4	
								BMI and FT3	
Ozsoy^[37]^	2018	Adults obese	Surgery	6 months	78 (49/29)	NA	Turkey	Weight and TSH	8/11
Ruiz-Tovar^[38]^	2013	Adults morbidly obese	Surgery	12 months	60 (7/53)	43.3 ± 10.4	Spain	Weight and TSH	8/11
Sari^[39]^	2003	Adults obese	Dietary modification	6 months	98 (0/98)	40.5 ± 11.4	Turkey	Weight and FT4	11/11
Svare^[40]^	2011	Adults	Dietary modification	120 months	11 720 (5066/9954)	NA	Norway	Weight and TSH	10/11
Yu^[41]^	2019	Adults obese	Surgery	12 months	65 (31/34)	39.9 ± 10.3	China	BMI and TSH	10/11

SD, standard deviation; BMI, body mass index; FT4, free thyroxine; TSH, thyroid-stimulating hormone; FT3, free triiodothyronine; JBI, Joanna Briggs Institute.

### Data extraction

Two researchers independently extracted data from the eligible papers by reviewing them in accordance with the study goals. The name of the first author, the year the study was published, the study’s location, the sample size, the type of intervention (dietary changes or surgery), the mean age, and the length of follow-up were among the extracted data. Furthermore, particular information on TH levels (TSH, FT3, and FT4) and anthropometric indices [body mass index (BMI), weight, waist circumference, and body fat percentage] was collected from the selected studies.

### Statistical analysis

Heterogeneity among the studies was assessed using Cochran’s *Q* test (significance level < 0.1) and *I*^2^ statistics (significance level > 50%). A random-effects model was applied for studies with significant heterogeneity, while a fixed-effect model was used for those without significant heterogeneity. To assess the sources of heterogeneity, subgroup analysis was conducted based on the type of intervention. Sensitivity analysis was performed to examine the influence of each individual study on the overall findings. Publication bias was evaluated using Egger’s regression test and visually assessed through funnel plots. The robustness of the results was further verified using trim-and-fill analysis. Prediction interval analysis was conducted to estimate the range of possible effects in future similar studies. Comprehensive Meta-Analysis Statistical Software (version 4) was utilized for all statistical analyses.

## Results

**Figure 1.**
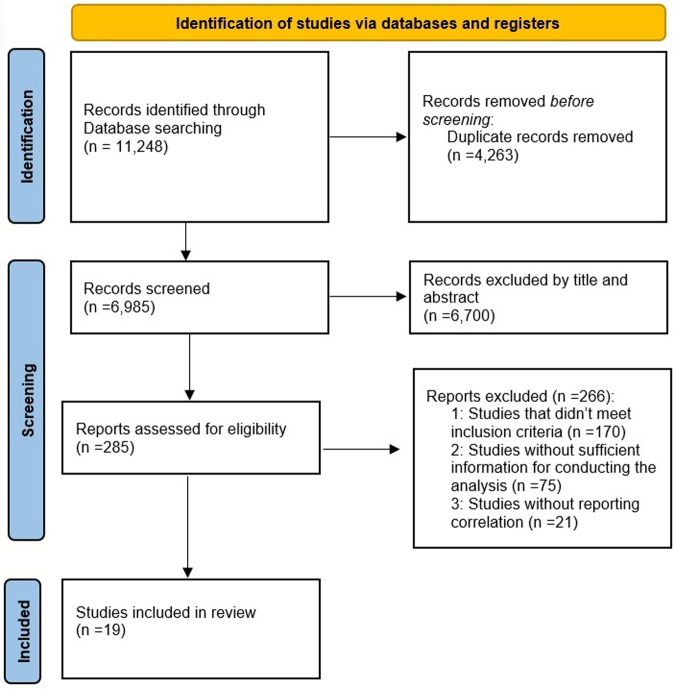
Study selection process.

Following the search of major databases, a total of 11 248 studies were initially identified. After removing 4263 duplicates, the titles and abstracts of the remaining 6985 papers were evaluated. Among these, 6700 studies were determined to be not relevant to the study objectives and were excluded. Subsequently, 285 studies proceeded to a full-text review. Ultimately, 19 studies met the inclusion criteria and were included in the final analysis. The selection process for these studies is illustrated in Figure 1.

**Figure 2.**
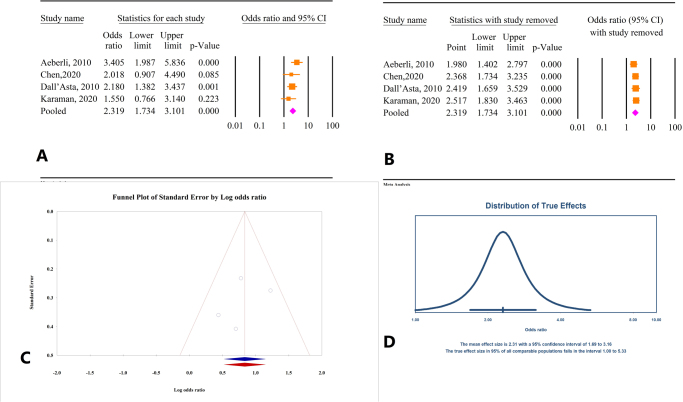
(A) Results of meta-analysis of the correlation between body mass index (BMI) and free triiodothyronine (FT3). (B) Results of sensitivity analysis. (C) Funnel plot of publication bias. (D) Results of prediction interval.

### Characteristics of the studies

Table 1 presents comprehensive data on the studies included in the meta-analysis. Of the 19 meta-analyses reviewed^[23–41]^, five were conducted in Turkey^[25,28,31,37]^, two in the USA^[27,34]^, two in Egypt^[23,30]^, two in China^[26,41]^, and one each in the Netherlands^[29]^, Switzerland^[24]^, South Korea^[32]^, Italy^[33]^, Brazil^[35]^, Palestine^[36]^, Spain^[38]^, and Norway^[40]^ (*N* = 19). The studies were conducted between 2003 and 2023, with sample sizes ranging from 20 to 11 720 participants. Among the 19 studies, 11 focused on surgical interventions^[23,25–27,29–31,35,37,38,41]^, while the remainder investigated dietary modifications. Only three studies specifically targeted children’s populations, with the rest focusing on adults.

### Result of meta-analysis

#### Correlation between decrease in BMI and FT3 changes

Based on the findings from four studies involving a total of 646 participants, a significant association was observed between a reduction in BMI and a decrease in FT3 levels [odds ratio (OR) = 2.31, 95% confidence interval (CI): 1.73–3.10, *P* < 0.01] (Fig. 2A). Sensitivity analysis indicated that the overall effect size remained stable even when each individual study was excluded (Fig. 2B), and there was low heterogeneity across the studies (*I*^2^ = 12.39%, *P* = 0.33). Assessment for publication bias using Egger’s and Begg’s tests showed non-significant results (*P* = 0.58 and *P* = 0.73, respectively). Trim-and-fill analysis confirmed the robustness of the results, with no additional studies (OR = 2.30, 95% CI: 1.68–3.15) (Fig. 2C). The prediction interval for the effect size ranged from 1.00 to 5.32 (Fig. 2D).

#### Correlation between decrease in BMI and FT4 changes

**Figure 3.**
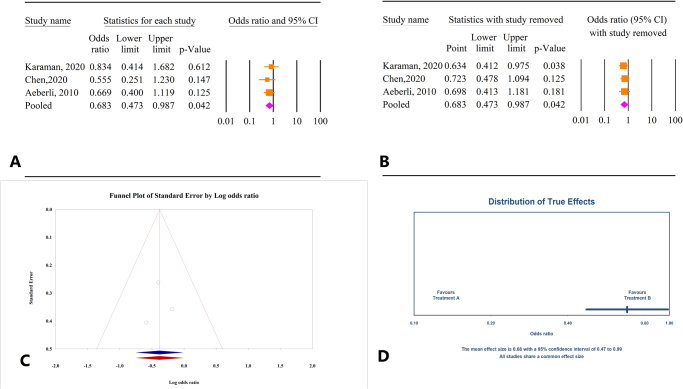
(A) Results of meta-analysis of the correlation between body mass index (BMI) and free thyroxine (FT4). (B) Results of sensitivity analysis. (C) Funnel plot of publication bias. (D) Results of prediction interval.

Based on the results of three studies involving a total of 388 participants, BMI reduction was associated with an increase in FT4 levels (OR = 0.68, 95% CI: 0.47–0.98, *P* = 0.04) (Fig. 3A). There was no significant heterogeneity among the studies (*I*^2^ = 0.0%, *P* = 0.75). Sensitivity analysis indicated that excluding the studies by Chen^[26]^ and Aeberli^[24]^ led to varying overall effects, resulting in non-significant outcomes (Fig. 3B). Tests for publication bias using Egger’s and Begg’s methods showed non-significant results (*P* = 0.91 and *P* = 1.0, respectively). Trim-and-fill analysis did not suggest the need for additional studies, confirming the consistency of the initial findings (OR = 0.68, 95% CI: 0.47–0.98) (Fig. 3C). The prediction interval for the effect size ranged from 0.47 to 0.99 (Fig. 3D).

#### Correlation between decrease in BMI and TSH changes

**Figure 4.**
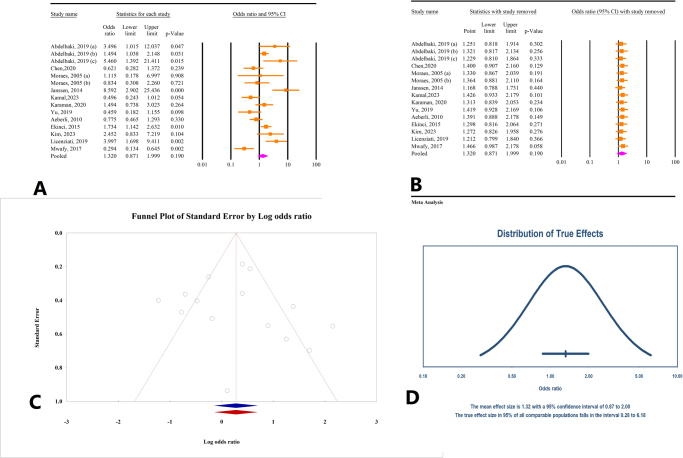
(A) Results of meta-analysis of the correlation between body mass index (BMI) and thyroid-stimulating hormone (TSH). (B) Results of sensitivity analysis. (C) Funnel plot of publication bias. (D) Results of prediction interval.

Analysis of data from 15 studies involving a total of 1553 participants revealed no significant correlation between decrease in BMI and TSH levels (OR = 1.32, 95% CI: 0.87–1.99, *P* = 0.19) (Fig. 4A). This finding was associated with substantial heterogeneity across the studies (*I*^2^ = 77.54%, *P* = 0.0). Sensitivity analysis demonstrated that excluding any individual study did not significantly alter the overall outcome (Fig. 4B). Tests for publication bias using Egger’s and Begg’s methods showed non-significant results (*P* = 0.74 and *P* = 0.27, respectively). Trim-and-fill analysis indicated no additional studies (OR = 1.31, 95% CI: 0.87–1.99) (Fig. 4C). The prediction interval for the effect size ranged from 0.28 to 6.18 (Fig. 4D).

#### Correlation between weight loss and FT3 changes

**Figure 5.**
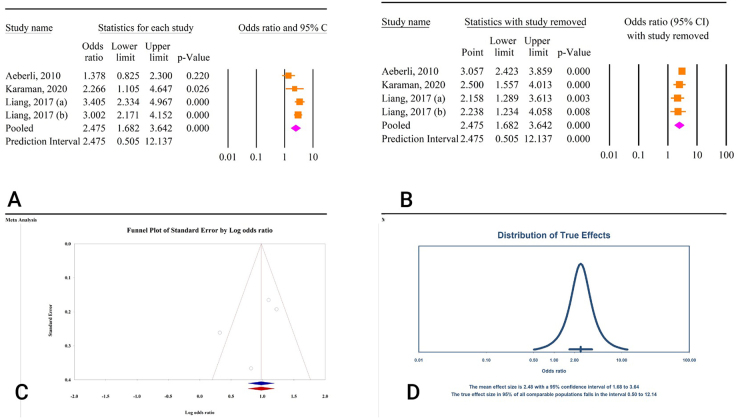
(A) Results of meta-analysis of the correlation between weight and free triiodothyronine (FT3). (B) Results of sensitivity analysis. (C) Funnel plot of publication bias. (D) Results of prediction interval.

From the analysis of four studies involving 1229 participants, weight reduction was associated with a decrease in FT3 levels (OR = 2.47, 95% CI: 1.68–3.64, *P* < 0.01) (Fig. 5A). Significant heterogeneity was observed among the studies (*I*^2^ = 64.95%, *P* = 0.03). Sensitivity analysis indicated that excluding any single study did not substantially change the overall effect (Fig. 5B). Publication bias tests using Egger’s and Begg’s methods did not show significant results (*P* = 0.35 and *P* = 0.73, respectively). However, trim-and-fill analysis suggested the addition of one study to adjust for potential publication bias, which produced consistent results (OR = 2.42, 95% CI: 1.44–3.30) (Fig. 5C). The prediction interval for the effect size ranged from 0.50 to 12.14 (Fig. 5D).

#### Correlation between weight loss and FT4 changes

**Figure 6.**
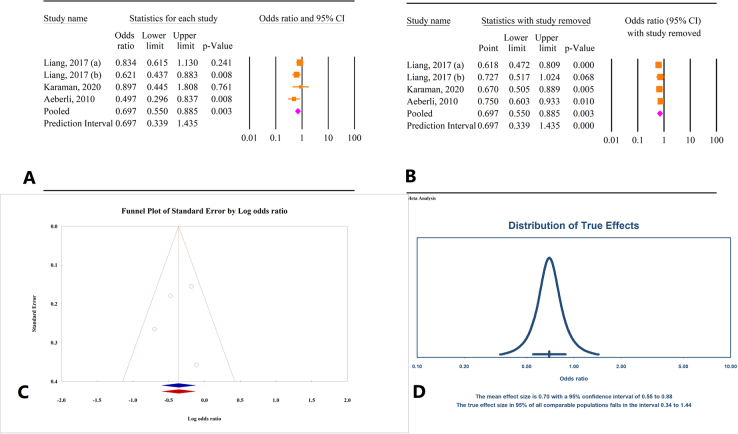
(A) Results of meta-analysis of the correlation between weight and free thyroxine (FT4). (B) Results of sensitivity analysis. (C) Funnel plot of publication bias. (D) Results of prediction interval.

Based on findings from four studies involving a total of 1275 individuals, a significant negative correlation was found between decrease in weight and FT4 levels (OR = 0.69, 95% CI: 0.55–0.88, *P* < 0.01) (Fig. 6A). There was no significant heterogeneity observed among the studies (*I*^2^ = 22.07%, *P* = 0.27). Sensitivity analysis showed a variation in the overall effect when the study by Liang *et al* (2017) was excluded (Fig. 6B). Tests for publication bias using Egger’s and Begg’s methods were not significant (*P* = 0.77 and *P* = 0.73, respectively). Trim-and-fill analysis did not suggest the need to include additional studies (OR = 0.69, 95% CI: 0.54–0.88) (Fig. 6C). The prediction interval for the effect size ranged from 0.34 to 1.44 (Fig. 6D).

#### Correlation between weight loss and TSH changes

**Figure 7.**
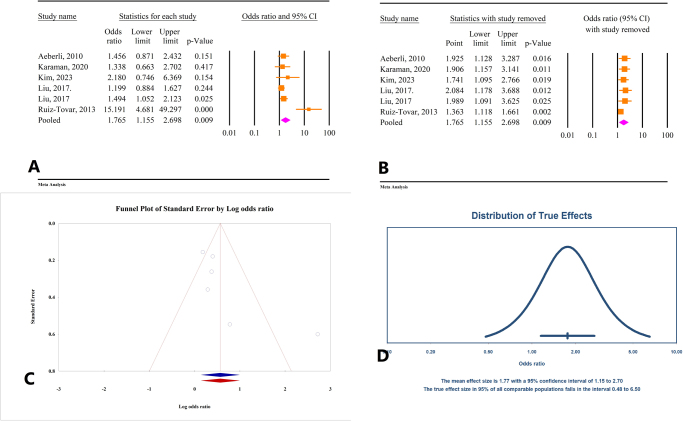
(A) Results of meta-analysis of the correlation between weight and thyroid-stimulating hormone (TSH). (B) Results of sensitivity analysis. (C) Funnel plot of publication bias. (D) Results of prediction interval.

The results from six studies involving a total of 1377 individuals indicated that weight loss leads to a decrease in TSH levels (OR = 1.76, 95% CI: 1.15–2.69, *P* < 0.01) (Fig. 7A). Significant heterogeneity was observed among the studies (*I*^2^ = 71.29%, *P* = 0.004). Sensitivity analysis demonstrated that the overall effect remained consistent when each individual study was excluded (Fig. 7B). Tests for publication bias using Egger’s and Begg’s methods were not statistically significant (*P* = 0.09 and *P* = 0.13, respectively). Trim-and-fill analysis did not indicate the need for additional studies, confirming the stability of the original findings (OR = 1.76, 95% CI: 1.15–2.69) (Fig. 7C). The prediction interval for the effect size ranged from 0.48 to 6.50 (Fig. 7D).

### Results of subgroup analysis based on intervention

**Figure 8.**
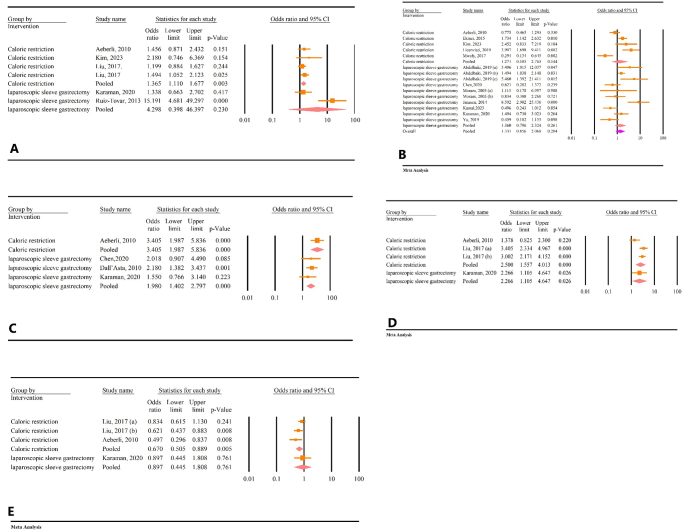
Subgroup analysis based on the type of intervention. (A) Weight and TSH. (B) BMI and TSH. (C) BMI and FT3. (D) Weight and FT3. (E) Weight and FT4.

The meta-analysis revealed a significant correlation between weight loss and TSH changes. Subgroup analysis based on the intervention type showed that this relationship remained significant for individuals undergoing calorie restriction (OR = 1.36, 95% CI: 1.11–1.67, *P* < 0.01, *I*^2^ = 0.00%). However, the correlation was not significant for patients who underwent laparoscopic bariatric surgery (OR = 4.29, 95% CI: 0.39–46.39, *P* = 0.23, *I*^2^ = 91.71%) (Fig. 8A).

The overall analysis of the correlation between decrease in BMI and TSH changes was not significant. This relationship remained insignificant for both the calorie restriction group (OR = 1.27, 95% CI: 0.58–2.76, *P* = 0.54, *I*^2^ = 85.34%) and the bariatric surgery group (OR = 1.36, 95% CI: 0.79–2.32, *P* = 0.26, *I*^2^ = 74.30%) (Fig. 8B).

Subgroup analysis demonstrated a significant association between BMI and FT3 changes in both the calorie restriction group (OR = 3.40, 95% CI: 1.98–5.83, *P* < 0.01, *I*^2^ = 0.00%) and the bariatric surgery group (OR = 1.98, 95% CI: 1.40–2.79, *P* < 0.01, *I*^2^ = 0.00%) (Fig. 8C).

Additionally, the subgroup analysis revealed a significant association between weight loss and FT3 changes for both the calorie restriction group (OR = 2.50, 95% CI: 1.55–4.01, *P* < 0.01, *I*^2^ = 76.41%) and the bariatric surgery group (OR = 2.26, 95% CI: 1.10–4.64, *P* = 0.02, *I*^2^ = 0.00%) (Fig. 8D).

Moreover, the subgroup analysis demonstrated a significant relationship between weight and FT4 changes in the calorie restriction group (OR = 0.67, 95% CI: 0.50–0.88, *P* < 0.01, *I*^2^ = 40.39%). However, this relationship was not significant in the laparoscopic bariatric surgery group (OR = 0.89, 95% CI: 0.44–1.80, *P* = 0.76, *I*^2^ = 0.00%) (Fig. 8E).

**Figure 9.**
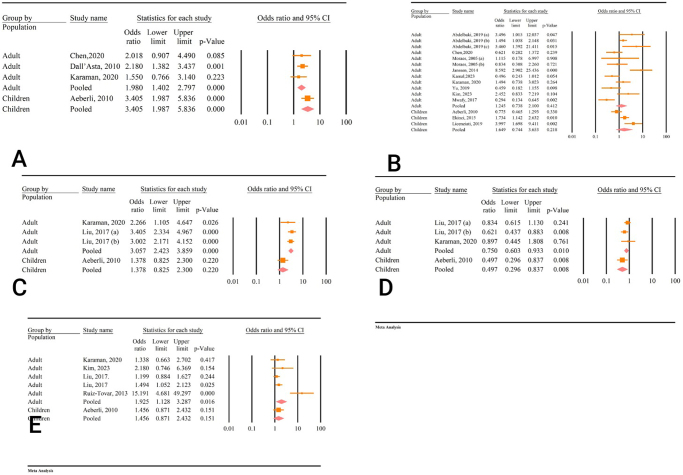
Subgroup analysis based on population. (A) Weight and TSH. (B) BMI and TSH. (C) BMI and FT3. (D) Weight and FT3. (E) Weight and FT4.

The results of the subgroup analysis based on population showed a significant correlation between BMI and FT3 changes in both adults (OR = 1.98, 95% CI: 1.42–2.79, *P* < 0.01) and children (OR = 3.41, 95% CI: 1.98–5.83, *P* < 0.01) (Fig. 9A). The correlation between BMI and TSH changes was insignificant in both adults (OR = 1.24, 95% CI: 0.73–2.10, *P* = 0.41) and children (OR = 0.64, 95% CI: 0.74–3.65, *P* = 0.21) (Fig. 9B).

A significant correlation between weight loss and FT3 was observed in adults (OR = 3.05, 95% CI: 2.42–3.85, *P* = 0.02), but not in children (OR = 1.37, 95% CI: 0.82–2.30, *P* = 0.22) (Fig. 9C). The correlation between weight loss and FT4 was significant in both adults (OR = 0.75, 95% CI: 0.60–0.93, *P* = 0.01) and children (OR = 0.49, 95% CI: 0.29–0.83, *P* < 0.01) (Fig. 9D). The correlation between weight loss and TSH was significant in adults (OR = 1.92, 95% CI: 1.12–3.28, *P* = 0.01), but not in children (OR = 1.45, 95% CI: 0.87–2.43, *P* = 0.15) (Fig. 9E).

## Discussion

The impact of thyroid function on body weight is extensively studied^[42]^. It is generally accepted that hyperthyroidism results in weight loss and hypothyroidism leads to weight gain^[43,44]^. However, the inverse relationship, specifically how weight loss affects TH levels, is less studied. The recent study by the Lancet Diabetes & Endocrinology Commission has declared that focusing only on weight and BMI-based measurements of obesity cannot fully elaborate on this chronic systemic issue^[45]^. Clarifying the possible correlations of obesity indices with each specific thyroid laboratory test across different populations and therapeutic approaches would elaborate the need for age-specific guidelines in managing thyroid function during weight loss. In this study, we assessed the impact of weight loss on TH levels by conducting a meta-analysis of longitudinal studies.

Our data showed that a decrease in weight and BMI is significantly correlated with TH levels. Specifically, a decrease in weight and BMI is positively correlated with FT3 and TSH, and negatively correlated with FT4. Previous studies on the impact of weight loss on THs have produced controversial results. Some studies reported a decrease in circulating TSH^[46–48]^ and FT3^[27,35,49]^ levels after metabolic surgery, while others did not confirm these findings^[27,50]^. In contrast with our results, the study of Lips *et al* declared the reduction in FT3 and TSH, regardless of the therapeutic intervention^[51]^. Additionally, some studies showed an increase^[27,52]^ in FT4 levels after bariatric surgery, while others reported no change^[35,47,53]^ or even a decrease^[49]^ in FT4 levels following the procedure. Aeberli’s study^[24]^, which involved obese children and adolescents and used dietary calorie restriction as the intervention, found that weight loss was significantly correlated with FT4 levels but had a non-significant correlation with FT3 and TSH levels. Regarding long-term follow-up, Reinehr *et al* reported sustained reduced levels of T3 and T4 (but not TSH) in obese pediatric who achieved weight reduction^[54]^. Our findings showed partial concordance with those reported by Zendel *et al* (2017), who conducted a study focused exclusively on bariatric surgery and thyroid function in obese patients^[55]^. However, a key point of divergence lies in TSH: while Zendel *et al* reported a significant postoperative reduction in TSH, our meta-analysis found no statistically significant effect of bariatric surgery on TSH levels^[55]^.

The variations among these studies may be attributed not only to the specific forms of metabolic surgery or diet regimens used but also to the baseline characteristics of the subjects, such as BMI, genetic differences in the study populations, the duration of follow-up, and the statistical methods employed^[26]^. Understanding these discrepancies is essential for tailoring interventions to optimize body composition and manage metabolic health outcomes in different populations.

To evaluate the potential role of different interventions, we implemented subgroup analyses based on the intervention type and divided the data into two major subgroups: one resulting from calorie restriction and the other from bariatric surgery. The findings showed a stronger association between weight loss and TH changes in the calorie restriction group compared to the bariatric surgery group. The distinct effects observed between calorie restriction and bariatric surgery interventions highlight the importance of intervention type in modulating TH levels.

In addition to the observed differences across intervention types, another important clinical issue requiring deeper consideration is the occurrence of SCH in obese patients^[23,56]^. SCH, characterized by elevated TSH levels with normal circulating THs (FT4 and FT3), is commonly encountered in obesity setting^[57–60]^. A growing body of evidence indicates that SCH arises as a result of excessive fat accumulation rather than serving as its underlying cause. Consequently, such elevation in TSH levels should often be considered an obesity-induced reversible condition rather than a chronic thyroid dysfunction^[61–63]^. Notably, several studies and clinical observations have shown that substantial weight reduction restores euthyroid status in obese patients diagnosed with SCH, challenging the necessity of lifelong levothyroxine (LT4) supplementation^[55,64]^. These findings carry important clinical implications. Clinicians should approach elevated TSH levels in obese patients with caution, emphasizing weight management strategies as the primary therapeutic intervention before resorting to pharmacological treatments. A misclassification of obesity-induced SCH as primary hypothyroidism could inadvertently lead to unnecessary LT4 therapy, posing potential risks. Therefore, individualized patient assessment, coupled with targeted weight reduction strategies, could optimize thyroid function management and reduce overtreatment. Future studies should prospectively evaluate the prevalence of SCH resolution following substantial and sustained weight loss, and examine whether distinct obesity phenotypes – such as central obesity, severe obesity, or metabolically unhealthy obesity – differentially impact the likelihood of thyroid function normalization.

The precise impact of weight loss on TSH and THs remains unclear. One perspective suggests that the enhancement of thyroid function is primarily due to weight loss itself, rather than solely the result of specific interventions like bariatric surgery^[65]^. This notion is supported by evidence indicating that weight loss from lifestyle changes can also lower TSH levels^[66]^. Additionally, these findings may be attributable to confounding factors including baseline nutritional status or hormonal effects of concomitant training exercises in the calorie restriction group^[67]^. However, to the best of our knowledge, no studies have directly compared the effects of bariatric surgery with lifestyle interventions on thyroid function. Understanding the distinct impacts of these interventions could provide valuable insights into the mechanisms underlying TH regulation and inform treatment strategies for individuals with thyroid dysfunction and obesity.

In addition to intervention-based subgroup analysis, we also conducted population-based subgroup analysis for adults and pediatrics. The main findings were mostly consistent across this subgroup analysis. However, it is noteworthy that despite the significant correlation between TSH and weight loss in adults, BMI reduction was not significantly related to TSH. This could be potentially attributable to the longitudinal design of included studies, resulting in heterogeneity and also differential reductions in fat and lean mass during weight loss in different studies^[68]^.

### Possible mechanism of actions

#### The impact of weight loss on TSH decrease

Various factors may contribute to alterations in thyroid function following weight reduction. When fat mass decreases, the release of leptin from fat cells also diminishes. This reduction can decrease stimulation of the hypothalamic–pituitary–thyroid (HPT) axis and hinder the conversion of T4 to T3 in the body. Consequently, there is a decrease in TSH and TH levels, which might be the primary mechanism responsible for the observed alterations in thyroid function^[69–71]^. Leptin, a hormone secreted by adipose tissue, helps control the activity of the thyrotropin-releasing hormone gene^[69]^.

Obesity can decrease the number of TSH receptors on the thyroid gland, making it less responsive to TSH and leading to lower production of THs. This can cause resistance to THs. However, losing weight can reverse this effect by increasing the number of TSH receptors, thereby improving the thyroid gland’s function and reducing TH resistance^[72]^.

Growth hormone also plays a key role in controlling the HPT axis and is much lower in obese individuals. Growth hormone increases during weight reduction and decreases TSH via the HPT axis^[65,73]^. Another possible mechanism is the reduction in body inflammation following weight loss, which has been linked to a decrease in TSH levels, leading to improved sensitivity to THs^[74]^.

Additionally, weight reduction has the potential to suppress the functioning of B and T lymphocytes and decrease the levels of thyroid autoimmune antibodies and inflammatory cytokines in the bloodstream. This might potentially safeguard the thyroid gland against inflammatory damage and the involuntary release of stored THs^[75]^. Moreover, weight loss has been shown to contribute to a reduction in TSH levels by improving glycemic and lipid profiles^[38,76]^.

**Figure 10.**
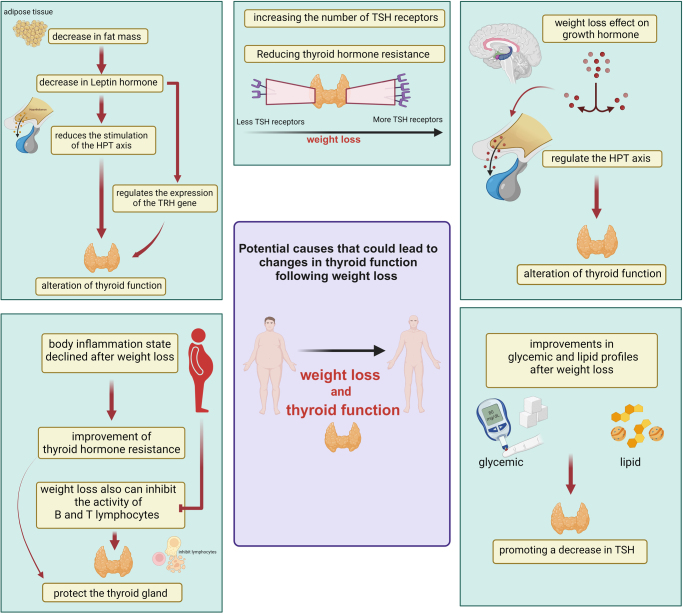
Possible mechanisms of action of the impact of weight loss on thyroid function.

Finally, TH binding to proteins impacts their total levels in circulation, and variations in protein binding ability (e.g., TBG levels) may influence total T3 and T4 concentrations^[11]^. These diverse mechanisms collectively highlight the complex relationship between weight loss and thyroid function, emphasizing the multifaceted impact of weight reduction on endocrine health (Fig. 10).

#### The impact of weight loss on FT4 increase

A significant inverse correlation between decrease in both BMI and weight and FT4 levels was found in our study. This finding might be a direct consequence of the loss of adipose tissue from the body, causing reduced redistribution of FT4 and thus elevated levels in blood circulation.

Another hypothesis is that weight loss increases the peripheral expression of TRs, thereby reducing TSH secretion through the suppression of the hypothalamic–pituitary–adrenal axis and decreasing the peripheral conversion of T4 to T3. This effect is attributed to the reduction in circulating leptin levels, which in turn reduces the activity of the deiodinase enzymes (predominantly D1 and D2) responsible for converting FT4 to FT3. This may be an adaptive response by the body to lower its metabolic demands and maintain optimal metabolic rates. Studies have described the relationship between deiodinase enzyme activity and weight loss, particularly when weight loss is abrupt or severe^[19,77,78]^ (Fig. 10).

#### The impact of weight loss on FT3

Our results have shown a positive and significant correlation of FT3 with decrease in both BMI and weight. This predicts that the levels of FT3 would be expected to reduce despite a rise in FT4 after weight loss. There are several hypotheses that support these findings. One of them, as mentioned earlier, is the impact of weight loss on deiodinases, leading to reduced peripheral conversion of FT4 to FT3^[19]^. Another hypothesis is that the total values of T3 may remain the same; however, the T3 synthesized is predominantly in a bound state. This could occur due to changes in physiological levels of plasma proteins after weight reduction, especially when achieved through lifestyle modifications. Higher levels of thyroglobulin-binding protein or albumin may reduce the levels of FT3 in the bloodstream. An important point to note is that a reduction in FT3 may not necessarily indicate reduced thyroid function, as TSH values have shown a reduction, implying a euthyroid state^[11]^. This suggests that the body may be adapting to maintain overall thyroid function and metabolic balance despite the changes in individual TH levels^[79]^ (Fig. 10).

### Strength, limitation, and future suggestions

In this study, we assessed the impact of weight loss on thyroid function by evaluating TSH, FT3, and FT4 levels. We also examined how calorie restriction and bariatric surgery influence THs differently and provided insights into the effects of weight loss on children and adolescents separately. A major strength of our meta-analysis is the inclusion of both surgical and non-surgical interventions across diverse age groups, enabling a broader understanding of endocrine responses to weight loss.

However, our study has several limitations. The number of included studies for each analysis is relatively low, and additional research with larger sample sizes is needed to confirm these findings. Although we performed subgroup analyses to explore the sources of heterogeneity, we could not fully account for all observed variations in the results. Another limitation is the lack of sex-specific analysis. Given the established physiological differences in TH metabolism between males and females, the absence of gender-stratified data limits the generalizability of our results across sexes. In addition, most of the original studies did not clearly report how many participants were on LT4 therapy. LT4 use can substantially alter serum TH levels, and without adequate stratification or reporting, it is difficult to determine whether observed hormonal changes reflect endogenous adaptation or are influenced by exogenous hormone replacement.

Future research should investigate the impact of follow-up duration, intervention type, ethnicity, and dietary approach on thyroid responses to weight loss. Studies should also assess the effects of comorbidities and evaluate newer pharmacologic interventions such as GLP-1 receptor agonists. Importantly, future trials and meta-analyses should stratify patients by LT4 therapy status to isolate its influence on TH trends. Furthermore, incorporating sex-specific analyses will provide greater insight into personalized management strategies for thyroid dysfunction in the context of obesity.

## Conclusion

The study results indicate that weight reduction leads to a decrease in TSH and FT3 levels and an increase in FT4 levels. Calorie restriction through diet has a more pronounced effect on thyroid function compared to bariatric surgery. Additionally, the decrease in FT3 does not indicate thyroid function imbalance, as TSH levels also decrease after weight loss.

Our findings underscore the necessity to explore various changes in patient care. It is essential to monitor and adjust treatment plans for hypothyroid patients undergoing weight loss. In addition, our study suggests that weight reduction could serve as a potential alternative to thyroxine supplements in treating the subgroup of patients with SCH and obesity.

## Data Availability

The datasets used and/or analyzed during the current study are accessible from the corresponding author on reasonable request.

## Research registration unique identifying number (UIN)

The study protocol was registered in PROSPERO with the registration code (CRD42024578894).
